# Influence of developmental stage on the antibiotic resistome and virome of the critically endangered kākāpō (*Strigops habroptilus*)

**DOI:** 10.3389/fmicb.2025.1654781

**Published:** 2025-10-21

**Authors:** Natalie Ayriss, Annie G. West, Rebecca K. French, Jemma L. Geoghegan, James Chatterton, Andrew Digby, Lydia Uddstrom, Kākāpō Recovery Team, Michael W. Taylor

**Affiliations:** ^1^School of Biological Sciences and The University of Auckland, Auckland, New Zealand; ^2^Institute of Ecology and Evolution, School of Biological Sciences, The University of Edinburgh, Edinburgh, United Kingdom; ^3^Department of Microbiology and Immunology, University of Otago, Dunedin, New Zealand; ^4^New Zealand Centre for Conservation Medicine, Auckland, New Zealand; ^5^Kākāpō Recovery Programme, Department of Conservation, Invercargill, New Zealand

**Keywords:** kākāpō, resistome, ARG, virome, metatranscriptomics

## Abstract

Endemic to Aotearoa New Zealand, the kākāpō is among the world’s rarest bird species with a current population of less than 250 individuals. As part of ongoing research efforts to support the conservation of this intensively managed species, we utilised a metatranscriptomics approach to elucidate, for the first time, the expression of antibiotic resistance genes within the kākāpō chick and adult gut microbiomes, further leveraging these data to describe the kākāpō virome. To determine differences among members of the kākāpō population, our data were obtained from birds encompassing different ages, sexes, geographic locations and antibiotic histories. We additionally analysed a time-series dataset following a single male kākāpō over the course of antibiotic treatment during a case of exudative cloacitis. There were significant differences between chicks and adult kākāpō in both the expression of antibiotic resistance genes and their viromes. Expression of these genes indicated potential resistance against 32 antibiotic classes, including 14 single classes of antibiotic and 18 multidrug classes. We identified 74 viral families, but no known avian-infecting viruses. Our case study of the single kākāpō during antibiotic treatment revealed notable changes in expression across time, with a reassuring lack of antibiotic resistance gene expression towards the end of the treatment, indicative of continued efficacy of antibiotic treatment. These novel data will help to inform conservation efforts for this enigmatic and unique bird species.

## 1 Introduction

Antibiotic resistance is a modern crisis with ancient origins. Now precipitated by the overuse and misuse of antibiotics in clinical and agricultural settings, resistance mechanisms initially evolved as part of a co-evolutionary arms race amongst competing bacterial species (Larsson and Flach, 2022). The emergence of antimicrobial compounds through secondary metabolites required the reciprocal development of diverse protective mechanisms such as efflux pumps, antibiotic target alteration and reduced permeability to antibiotics that are still observed today (Christaki et al., 2020). As an acknowledged, potentially existential threat to human health, research into antibiotic resistance genes (ARGs) has understandably focused primarily on the “clinical resistome,” with comparatively little attention given to the potential consequences of such resistance for wildlife, including threatened species (Ramey and Ahlstrom, 2020). Nonetheless, existing research to date has unequivocally shown that wildlife populations are indeed sources of ARG diversity (Vittecoq et al., 2016; Li et al., 2024), with resistance documented across a vast array of wild species–from European carnivores (Garcês and Pires, 2023) to African grazers (King and Schmidt, 2017) and Australian pinnipeds (Fulham et al., 2022). A study into wild migratory birds recorded >1030 ARGs and 202 resistance types across 10 species (Cao et al., 2020). Furthermore, animals at the interface of urbanisation or exposed to anthropogenic environmental contaminants are more likely to encounter human-associated ARGs, creating potential reservoirs within natural ecosystems. Birds feeding at wastewater treatment plants, for example, express a number of resistance genes within their metatranscriptomes (Marcelino et al., 2019).

Among wildlife, threatened species may be especially vulnerable to the risks posed by antibiotic-resistant bacteria (West et al., 2019; Fulham et al., 2022; Ienes-Lima et al., 2023). Even with limited use of antibiotics in managed threatened species, the risk of ARG acquisition remains a valid concern. While vertical gene transfer through microbial reproduction increases the risk of ARG transmission, horizontal gene transfer is particularly concerning, with mobile genetic elements such as plasmids and transposons capable of transmitting ARGs between bacterial species (Arnold et al., 2022). With a lack of viable alternative treatments for combating future antibiotic-resistant infections, the vulnerability of endangered species may be further exacerbated, leading to greater population decline or even extinction. The critically endangered kākāpō (*Strigops habroptilus*) is one such species for which the theoretical risk of antibiotic resistance could prove consequential.

A flightless parrot that is endemic to Aotearoa New Zealand, the kākāpō is among the world’s rarest and most unique bird species. Once the second most abundant bird species in the New Zealand Holocene according to the fossil record (Boast et al., 2025) it is now extinct in its natural range. The entire population [approximately 245 individuals, up from a low of 51 in 1995 (Powlesland, 2006)], is intensively managed by the New Zealand Department of Conservation’s Kākāpō Recovery Programme and is confined to a handful of predator-free sanctuaries across the country. The conservation effort for kākāpō is complicated by infrequent breeding seasons and low hatching success (Powlesland and Lloyd, 1994; Savage et al., 2021), the ongoing threat of invasive mammalian predators precluding a return to their natural range, and the recent emergence of kākāpō diseases such as erysipelas, aspergillosis and exudative cloacitis (Gartrell et al., 2005; Jakob-Hoff and Gartrell, 2011; Winter et al., 2022; West et al., 2025). The treatment of such diseases in kākāpō necessitates varying degrees of veterinary intervention, including the frequent use of antibiotics. The two most commonly used antibiotics in kākāpō are enrofloxacin (a fluoroquinolone) and an amoxicillin-clavulanate combination (a penicillin and beta-lactamase inhibitor) (J. Chatterton, L. Uddstrom, pers. obs.). The use of antibiotics in such a threatened species represents a potential double-edged sword whereby treatment is beneficial in the short term but conceivably contributes to ARG proliferation in the longer term, perhaps hindering future treatments in a world where the threat from disease is only likely to increase (West et al., 2019). While treatment of kākāpō remains largely effective, there exists anecdotal evidence for some level of antibiotic resistance within the population: specifically, in *Escherichia coli* cultivated from an individual which had previously received antibiotic treatment for aspergillosis (J. Chatterton, pers. obs.). Given this specific observation, within the broader context of general concerns around antibiotic resistance, we sought to apply a metatranscriptomic approach to determine whether ARGs are actively expressed by the microbial community of the kākāpō gastrointestinal tract. Metatranscriptomics enables not only the identification of ARG types but also quantification of their expression, as shown previously for chickens and other animal microbiomes (Wang et al., 2020). Yet another benefit is that the data can be interrogated for the presence of RNA viruses (Chang et al., 2021; Luo et al., 2025). Two recent metatranscriptomic studies attempted to identify a potential viral cause for exudative cloacitis in kākāpō (West et al., 2025; French et al., 2025b), though the exact cause for this disease remains equivocal. Another application of this technology indicated the presence of a low-diversity virome in adult kākāpō (French et al., 2023, 2025a), though the pooling of samples precluded the collection of individual-level data. There is currently no information on viruses found in kākāpō chicks, or the subsequent development of the kākāpō virome as birds age.

In this metatranscriptomics study, we took a three-pronged approach to analyse cloacal swab samples obtained from kākāpō living on two separate islands. Firstly, we focused on individuals from the general adult kākāpō population according to their antibiotic history, i.e., individuals which had received previous antibiotic treatment at any given time versus those which had not. Secondly, we analysed pooled samples from antibiotic-naïve chicks in a longitudinal time series to document changes in the resistome as the chicks developed. Finally, we investigated ARG expression in a single adult male kākāpō over the course of antibiotic treatment for exudative cloacitis. Data from the individual adults and pooled chicks were further leveraged with the aim of establishing the kākāpō virome.

## 2 Materials and methods

### 2.1 Sampling

All adult and chick samples were collected from kākāpō living on Whenua Hou/Codfish Island (46°77’S, 167°63′E) or Anchor/Pukenui Island (45°76’S, 166°51′E) during routine health checks by Department of Conservation staff throughout 2022. Fourteen samples were obtained from adults, of which six had historically been treated with antibiotics and eight had not. Samples from antibiotic-naïve chicks were collected at four time points for later pooling of extracted RNA. This pooling strategy was applied due to anticipated low amounts of RNA available to be extracted from an individual chick swab, as well as budgetary considerations. Samples were also collected from one adult individual (“Joe”) at five timepoints over the course of 11 days of antibiotic treatment. The initial sample from this individual was collected pre-treatment, then at 1, 2, 8, and 10 days into antibiotic treatment including enrofloxacin, amoxicillin-clavulanate and ceftiofur. For simplicity, the metadata and pooling information for these samples is confined to the Supplementary Tables S1, S2. All samples were obtained via gentle insertion of a flocked swab (Copan Diagnostics, USA) into the cloaca of the bird, followed by gentle rotation. Swabs were immediately placed into sterile 5 mL screw-cap tubes filled with 2.5 mL of RNA*later* for short-term storage at −20 °C, then chilled on ice during transit to the University of Auckland and subsequently frozen at −80 °C until RNA extraction was performed.

### 2.2 RNA extraction and sequencing

Total RNA was extracted from swabs using the RNeasy Plus Mini Kit and QIAshredders (Qiagen), following the protocol of French et al. (2023). Briefly, each tube containing a swab was thawed on ice, with sterilised forceps used to remove the swab into a sterile screw-cap polypropylene tube containing 600 μL of extraction buffer. The screw-cap tube was vortexed at maximum speed for 2 min. The swab and buffer were then removed, placed in a QIAshredder and centrifuged at 21,000 RCF for 5 min. The flow-through was retained and used as instructed in the RNeasy protocol. RNA was eluted into 60 μL of sterile nuclease-free water. A further DNase treatment was performed using the TURBO DNA-free™ Kit (Invitrogen) and a 55-cycle 16S rRNA gene-targeted PCR with gel visualisation was performed to check extractions for DNA contamination. Clean-up and concentration of RNA was performed using the NucleoSpin RNA Clean-up XS, Micro kit (Macherey-Nagel). The concentrated RNA was eluted into 30 μL of sterile nuclease-free water. For chicks, RNA was extracted from up to eight individuals per island at each time point, then pooled into a single library at equimolar concentration, to a total volume of 30 μL (Supplementary Table S2).

Library preparation and sequencing were performed by Auckland Genomics Ltd. (University of Auckland, New Zealand). Total RNA (100 ng) was input to the Illumina® Stranded Total RNA Prep with Ligation, Ribo-Zero (Illumina, cat 20040529) protocol to deplete ribosomal RNA, then ligation-based addition of adapters and indexes (IDT® for Illumina® DNA/RNA Unique dual indexes, Set D) was used to make stranded libraries for Illumina NovaSeq 6000 sequencing. Two S1 300 cycles (2 × 150 paired end) of the V1.5 kit were used, yielding a total of 1.268 TB of data and 2,010,840,192 reads.

### 2.3 Sequence data processing

Raw forward and reverse sequence reads from both NovaSeq runs were concatenated for each library and checked for quality using FastQC (v.0.11.9; Andrews, 2010). Trimmomatic (v.0.39; Bolger et al., 2014) was used to trim and filter reads; the remaining sequences were then aligned against a masked kākāpō genome (NCBI bStrHab1_v1. p, GCF_004027225.2) using BBMap (v. 39.01; Bushnell, 2014) to remove host-mapped RNA from the dataset. Further processing was performed to remove rRNA reads with SortMeRNA (v. 4.3.6; Kopylova et al., 2012).

The remaining non-rRNA from the SortMeRNA output was used for *de novo* assembly into transcripts using rnaSPAdes (v.4.0.0; Prjibelski et al., 2020). Assembly statistics were obtained using the “stats.sh” function of BBMap.

#### 2.3.1 Antibiotic resistome

To analyse the antibiotic resistome, contigs <1000 bp were considered too short for robust gene annotation and removed from the assemblies (He et al., 2021). The “dedupe.sh” function (minidentity = 100) from BBmap was used to produce a single set of dereplicated contigs for downstream processes and a read mapping index was generated from this. Reads filtered against the masked kākāpō genome were mapped to the index (ambiguous = best, minid = 0.95) and SamTools (v. 1.10; Danecek et al., 2021) was used to sort and convert the resulting SAM files to BAM format. Prodigal (v. 2.6.3; Hyatt et al., 2010) was used to identify protein coding sequences which were annotated with the Comprehensive Antibiotic Resistance Database (CARD) rgi (v. 5.2.0; Alcock et al., 2023). The Antibiotic Resistance Ontology (ARO) terms within CARD are standardised categories used to describe specific antibiotic resistance mechanisms and genes. Each ARO term classifies resistance mechanisms related to particular classes of antibiotic, providing a framework to compare transcripts and analyse resistance profiles in microbial communities. Annotations from CARD were only included for further analysis if they had at least 35% identity to an ARO in the database (Rost, 1999) and 70% query coverage, with “loose” hits not retained. A gene-coordinates SAF file was generated from the Prodigal protein prediction output and mapped reads were assigned to this via the “featureCounts” function of Subread (v. 2.0.3; Liao et al., 2014). Gene-level coverage statistics were generated based on this output, using a custom python script (summarise_counts.py, available at https://github.com/GenomicsAotearoa/environmental_metagenomics), and counts were normalised by transcript per million (TPM) to accommodate differences in gene length.

#### 2.3.2 Virome

The full transcript assembly was used for viral work, as RNA virus genomes can be small and useful information may exist in short fragments. In brief, viruses were identified using VIBRANT (v. 1.2.1; Kieft et al., 2020), VirSorter2 (v. 2.2.3; Guo et al., 2021) and through comparison of the assembled contigs to the National Center for Biotechnology Information (NCBI) nucleotide database (nt) using Blastn (BLAST+ v 2.9.0; Camacho et al., 2009). Contigs were further compared to the NCBI non-redundant protein database (nr) using Diamond Blastx (Diamond v. 2.1.10; Buchfink et al., 2015) with a maximum expected value (e-value) threshold of 1 ×10^−10^ as a significance cut-off. Contigs containing significant hits to bacteria or eukaryotes were removed, as were those matching to previously identified viral contaminants (Asplund et al., 2019).

Diamond BlastX was also performed against a custom database containing RNA dependent RNA polymerase (RdRp) sequences obtained from NCBI, using an e-value cut-off value of 1 × 10^−5^. A custom python script (virome_per_sample_derep.py, available at https://github.com/GenomicsAotearoa/environmental_metagenomics) was used to dereplicate outputs per assembly and contigs were assessed for quality using CheckV (v. 1.0.1; Nayfach et al., 2021). Further dereplication of putative viral contigs across all assemblies was performed to generate viral OTUs using a cluster-based method (Roux et al., 2017). A read mapping index was generated from these contigs using BBmap. Reads were mapped to this index (ambiguous = best, minid = 0.95), then SamTools used to sort and convert SAM files to BAM format. Summaries of contig-level coverage were generated using the summarise_counts.py script.

### 2.4 Quantitative and statistical analyses

All statistical and quantitative analyses were conducted in R (v. 4.3.2; R Core Team, 2023), with data visualisation and plotting performed using the R package ‘ggplot2’ (v. 3.4.3; Wickham, 2016). Phyloseq objects from the ‘phyloseq’ R package (v 1.34.0; McMurdie and Holmes, 2013), typically used for microbiome (microbial taxa) analyses, were repurposed to aggregate antimicrobial resistance features (ARGs, resistance mechanisms, drug classes and ARO terms) at their respective hierarchical levels. Phyloseq objects were also used for viral taxa. For Kruskal-Wallis comparisons between groups, post-hoc Dunn’s tests were carried out using the ‘dunn.test’ R package (v. 1.3.6, Dinno, 2017) with Holm *p*-value correction.

The “vegdist” function of the ‘vegan’ R package (v. 2.6.4; Oksanen et al., 2020) was used to calculate dissimilarity matrices based on Bray–Curtis dissimilarity for subsequent ordination using non-metric multidimensional scaling (nMDS). Ellipses were drawn around the data points using the “stat_ellipse” function of ggplot2 with assumed multivariate t-distribution. The “adonis2” function of the ‘vegan’ package was then applied to distance matrices to obtain permutational multivariate analysis of variance (PERMANOVA) results. All PERMANOVA analyses used 999 permutations unless stated otherwise. The “betadisper” function of the ‘vegan’ package was used to test for homogenous group dispersion.

Due to missing or replaced individuals within the pooled chick libraries across timepoints, these data were treated as pseudo-repeated measures. Linear mixed modelling (LMM) was conducted using the “lmer” function of the ‘lme4’ R package (v. 1.1.35.1; Bates et al., 2015), to determine the significance of aging across chick samples while accounting for the random effects of the different islands using the random intercept (1 | Island). Significance of the models was tested using likelihood ratio tests comparing the target model against a null model with ANOVA along with the “confint” function of the ‘stats’ package.

## 3 Results

### 3.1 Kākāpō metatranscriptome libraries

**Figure 1:**
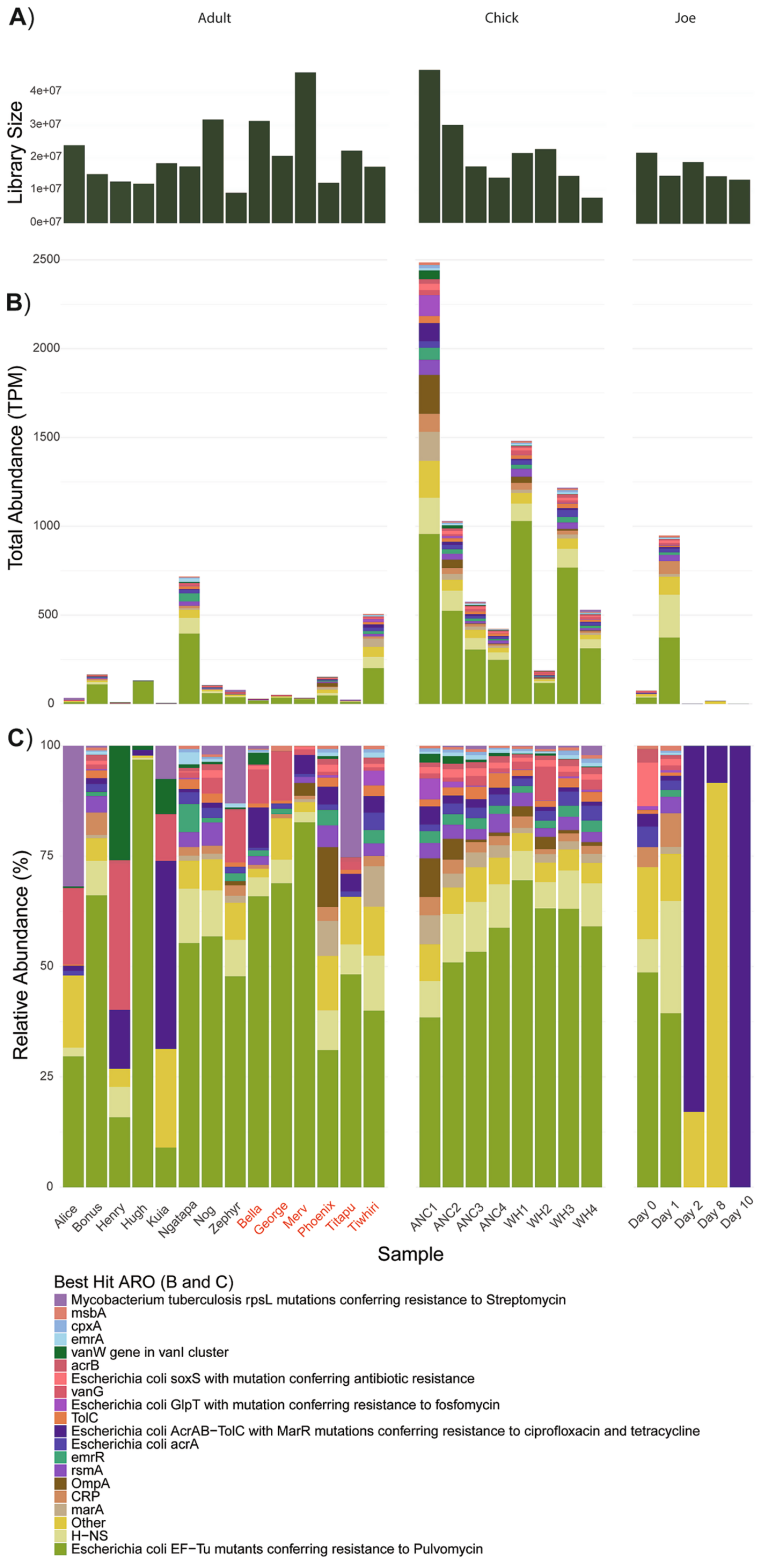
**(A)** Library sizes following removal of host-derived and rRNA sequences; **(B)** Total abundance of ARG transcripts; **(C)** Relative abundance of ARG transcripts. The “Other” category represents AROs that were expressed < 58 transcripts per million (TPM). Data grouped by adults, chicks and antibiotic case-study Joe, where day 0 represents pre-treatment. Adult kākāpō are referred to by name with previous antibiotic exposure indicated by red text. ANC refers to Anchor Island, WH refers to Whenua Hou Island.

A total of 27 kākāpō metatranscriptome libraries (encompassing 27 individual or pooled RNA extracts from cloacal swab samples; Supplementary Table S2) were sequenced. An average of 128 million raw sequence reads were obtained from each library at a range of 42 and 317 million raw reads. Removal of kākāpō host sequences and ribosomal RNA resulted in an average library size of 20 million reads (ranging from 7 to 46 million reads) (Figure 1A). There was no significant difference in median library size (and therefore sequencing depth) of individual adult kākāpō compared with either pooled chick samples or the treatment case study (Joe); this held true for both the raw reads and for reads remaining after removal of rRNA and host sequences (Kruskal-Wallis rank-sum test; *p* = 0.90, *p* = 0.82 respectively). Similarly, library size did not systematically influence recorded ARG expression or distribution of viruses (Spearman’s rank correlation tests; *p* = 0.44, *p* = 0.59).

### 3.2 The antibiotic resistome of the kākāpō

#### 3.2.1 Antibiotic resistome in the kākāpō population

**Figure 2:**
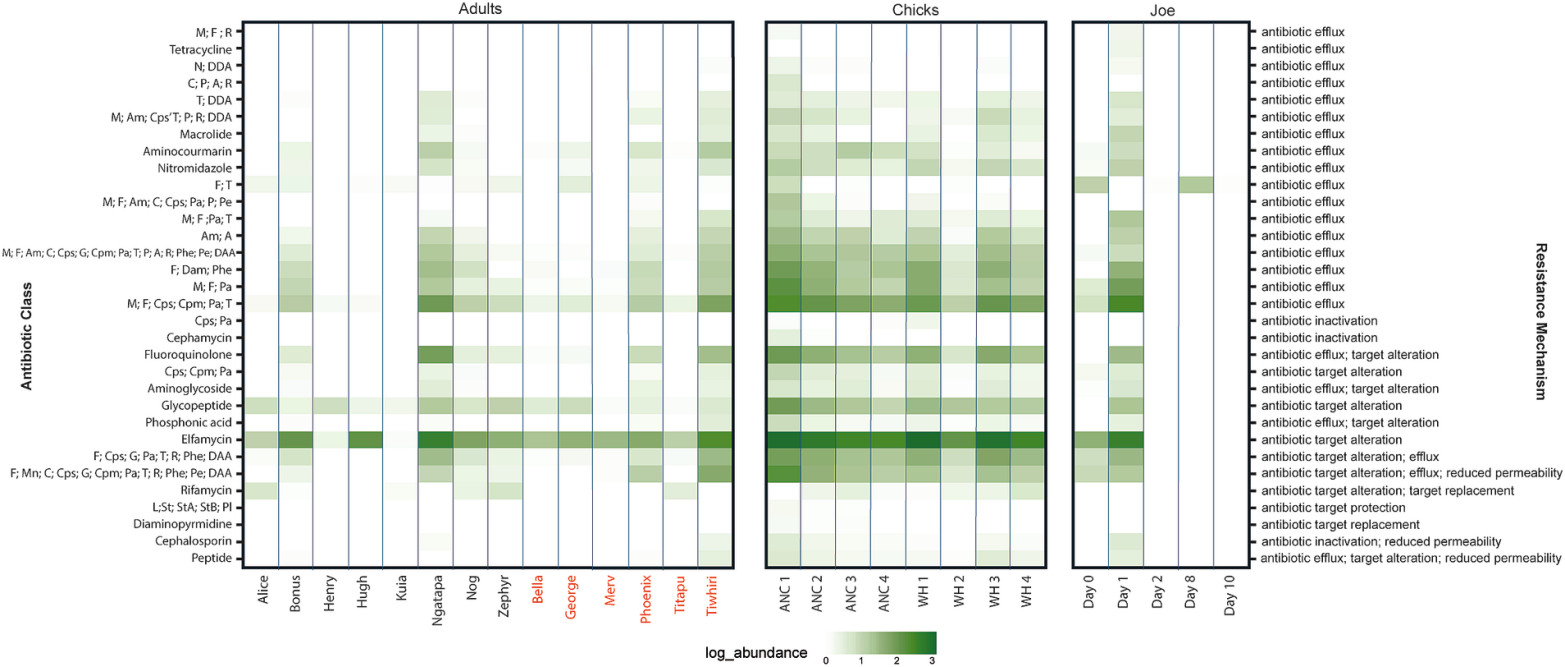
Heat map of log relative abundance (%) of transcripts classified by resistance mechanism (right axis) and drug class (left axis). Kākāpō with previous antibiotic exposure indicated by red text. For multidrug classes, a letter code is used to represent specific antibiotic types: A (aminocoumarin antibiotic), Am (aminoglycoside antibiotic), C (carbapenem), Cpm (cephamycin), Cps (cephalosporin), DAA (disinfecting agents and antiseptics), E (elfamycin antibiotic), F (fluoroquinolones), G (glycylcycline), Gp (glycopeptide antibiotic), L (lincosamide antibiotic), M (macrolide antibiotic), Mn (monobactam), N (nucleoside antibiotic), Ni (nitroimidazole antibiotic), Pa (penam antibiotic), Pe (penem antibiotic), P (peptide antibiotic), Phe (phenicol antibiotic), Pho (phosphoric acid antibiotic), Pl (pleuromutilin antibiotic), R (rifamycin antibiotic), St (streptogramin antibiotic), StA (streptogramin A antibiotic), and StB (streptogramin B antibiotic).

Overall, across the sampled adult and chick kākāpō cohorts we identified 515 expressed genes associated with antibiotic resistance. These were in turn classified into 65 unique Antibiotic Resistance Ontology (ARO) terms, which encompass various resistance mechanisms and confer resistance to a range of antibiotics. No single ARG was found exclusively in one kākāpō. Expression of these genes and their corresponding AROs indicates potential resistance against 32 antibiotic classes, including 14 single classes of antibiotic and 18 multidrug classes. The most prevalent resistance mechanism against these classes was antibiotic efflux, included in 23 of the identified antibiotic drug resistances (Figure 2). Antibiotic target alteration was another common resistance mechanism, associated with 10 different antibiotic drug classes (including multidrug resistance) (Figure 2). One particular ARO, “*Escherichia coli* EF-Tu mutants conferring resistance to pulvomycin,” was detected universally across both adult and chick kākāpō at high levels of expression (Figures 1B,C). This was the most abundantly expressed ARO in 19 of the 27 adult and chick libraries and is associated with resistance to elfamycin antibiotics, in particular pulvomycin. The second most expressed ARO differed among individual kākāpō, however for 10 samples this was observed as “histone-like nucleoid structuring protein” (H-NS). Additionally, H-NS was observed in all kākāpō except Kuia. H-NS is associated with multidrug resistance against five different classes of antibiotics including penicillin beta-lactam, tetracycline, cephalosporin, fluoroquinolone, and macrolide antibiotics. For two of the three kākāpō from Whenua Hou which had previous exposure to antibiotics, “*E. coli* AcrAB-TolC with MarR mutations” was the second most highly expressed ARO after the *E. coli* EF-Tu mutants. This ARO was detected in all sampled kākāpō except George and Zephyr.

**Figure 3:**
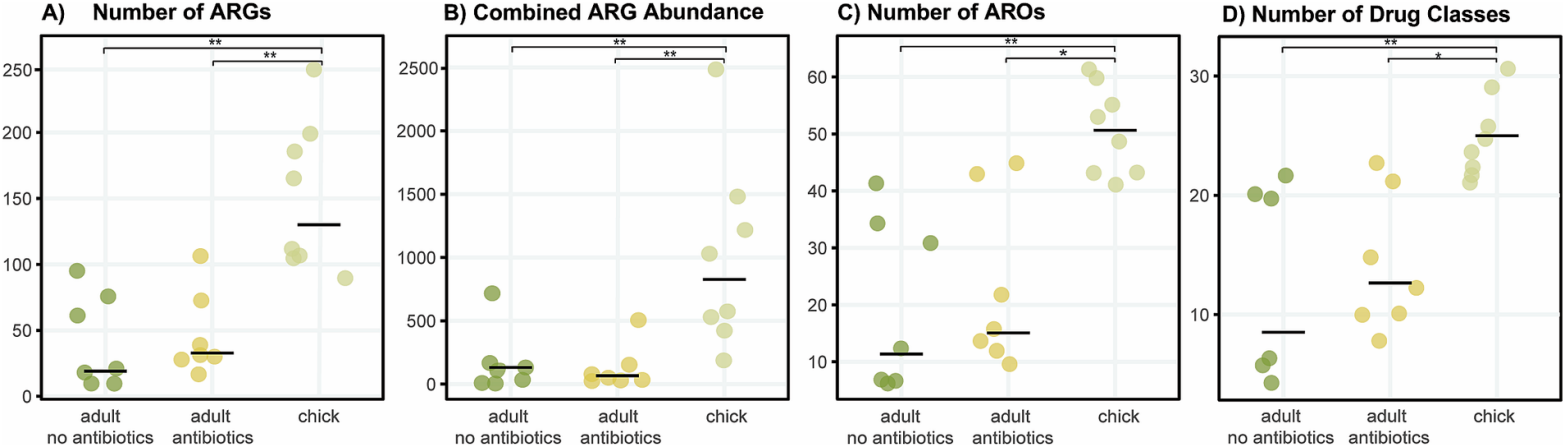
Diversity and abundance of ARGs and associated AROs and drug classes across kākāpō of different age groups and antibiotic history. For adults, each dot represents a library from a single individual kākāpō sample, whereas for chicks each dot represents a library pooled from samples of multiple individuals (see Supplementary Table S1). No chicks had ever received antibiotic treatment prior to their sampling for this study. Horizontal bars indicate median values. **(A)** Number of antibiotic resistance genes; **(B)** abundance of all expressed resistance genes in transcripts per million; **(C)** number of AROs; **(D)** number of drug classes (including multidrug resistances of combined classes). Kruskal-Wallis rank sum tests, ***p < 0.001, **p < 0.01, *p < 0.05.

Compared to adults, chicks had a higher median number of ARGs, greater combined ARG abundance (expression), increased diversity of AROs and resistance to a greater number of antibiotic classes (including multidrug resistance categories) (Figure 3). In most cases, adult kākāpō with a history of antibiotic exposure displayed slightly (but non-significantly) increased values for each of these categories compared with their antibiotic-naïve counterparts, with the exception of combined ARG abundance. Significant differences were found between all three adult antibiotic-naïve and antibiotic-exposed kākāpō and antibiotic-naïve chick groups (Kruskal-Wallis, *p* < 0.05). The post-hoc Dunn’s tests confirmed in each case that significant differences existed between chicks and adults of both statuses while no significant differences were observed between the adults of differing antibiotic history.

**Figure 4:**
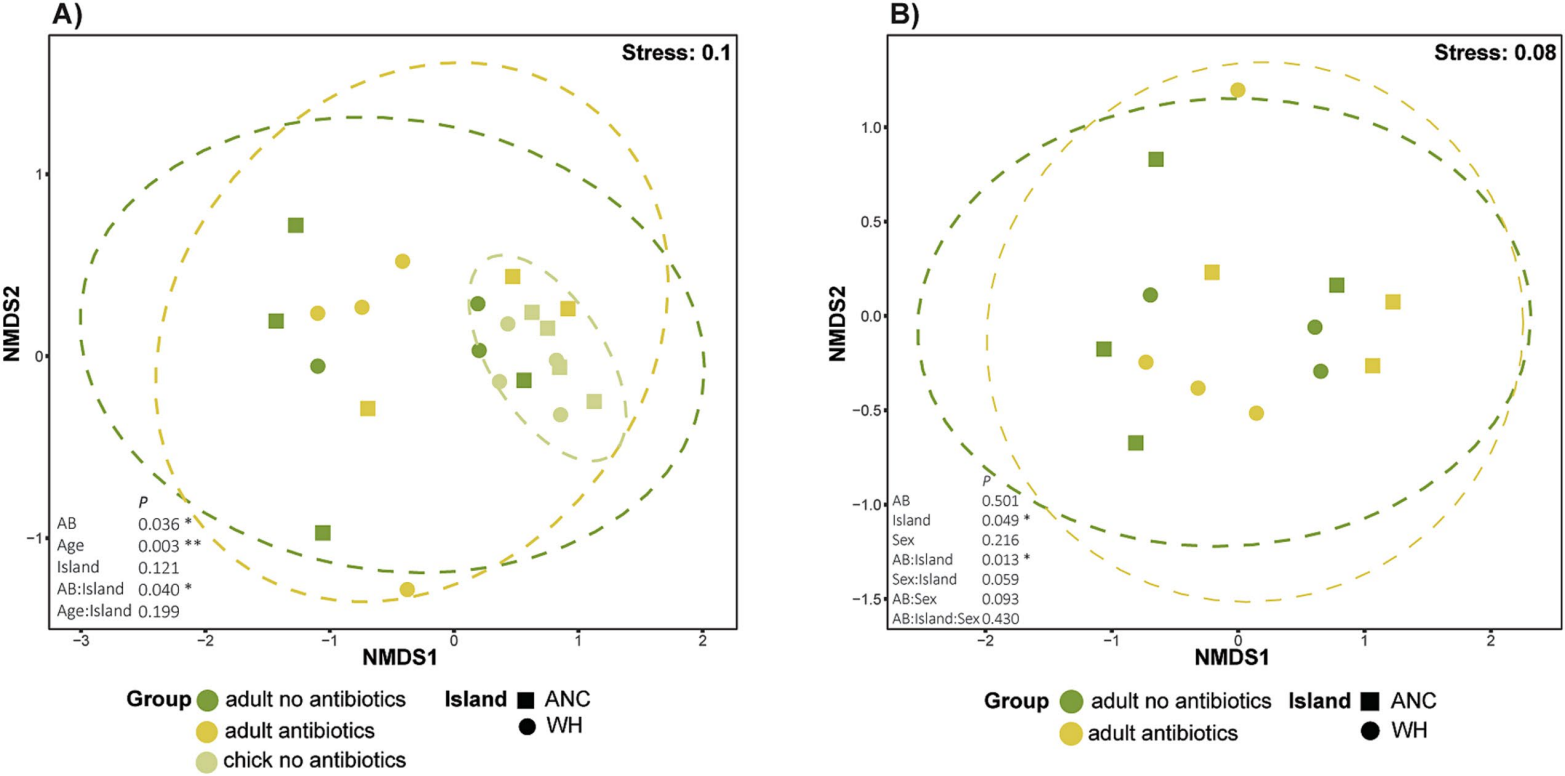
Non-metric multidimensional scaling (nMDS) ordination based on Bray–Curtis dissimilarity, representing resistomes for **(A)** kākāpō adults and chicks according to previous antibiotic exposure; **(B)** adult kākāpō only. Each point represents a unique sample. Adult kākāpō are individuals, whereas chick samples are pooled. Ellipses represent variance observed amongst each sampling group, with 95% confidence intervals for each sampling group. PERMANOVA results are shown as *p* values.

Pooled chick resistomes (Supplementary Table S2) exhibited much less dispersion among samples than either adult antibiotic grouping, clustering closely on the nMDS ordination while some overlap was observed between adults of both antibiotic statuses (Figure 4A). Antibiotic history (PERMANOVA, *R*^2^ = 0.07963, *p* = 0.036) and age (*R*^2^ = 0.15235, *p* = 0.003) both significantly influenced resistome composition, with bird age accounting for ~15% of resistome variation. While geographic location (island) did not significantly affect resistome composition, the interaction between antibiotic history and island was significant (*R*^2^ = 0.07978, *p* = 0.040). Despite the tight clustering of chick samples, there were no significant differences detected in homogeneity of dispersion (betadisper), suggesting that observed differences in beta-diversity were not driven by unequal dispersion among groups. Pairwise analysis of the interaction between antibiotic history and island revealed significant differences between kākāpō on both Anchor and Whenua Hou islands that had not received antibiotics compared to those on Whenua Hou that had (*p* < 0.05); however, adjustment of *p-*values (to account for multiple comparisons) rendered these comparisons no-longer statistically significant.

A subsequent analysis that excluded chicks allowed further investigation into the effects of various factors including sex on the resistomes of adult kākāpō (Figure 4B). Neither sex nor antibiotic status significantly influenced resistome beta-diversity. By contrast, the effect of geographic location (island) was marginally significant (*R*^2^ = 1.91, *p* = 0.049), with the interaction between antibiotic status and island again identified as significant (*R*^2^ = 2.8305, *p* = 0.013). Pairwise analysis in this instance showed that the most significant difference was between individuals that had not received antibiotics on Anchor Island and Whenua Hou. Regardless, and similarly to the previous analysis, adjusted *p*-values did not meet the threshold of significance. Once more, the betadisper test showed no significant differences in dispersion.

#### 3.2.2 Resistome changes with chick developmental stage

Chicks from Anchor Island had higher overall expression of ARGs at the first time point compared to those from Whenua Hou, with a subsequent decline in expression at each time point (Figure 1B). Chicks from Whenua Hou showed a sharp decrease in expression between the first and second time points, then another increase in expression by the third time point with a final decrease by the fourth time point. Linear mixed modelling revealed that while total ARG expression by TPM significantly decreased in chicks between 4 and 20 weeks of age (*X^2^* = 10.301*, p* = 0.026), the number of different ARGs, number of AROs and number of drug classes did not significantly decrease.

#### 3.2.3 The resistome of an individual kākāpō during antibiotic treatment

While statistical analyses could not be undertaken for this portion of the project with treatment being limited to a single individual (Joe), we could nonetheless make some useful observations of resistome changes during antibiotic treatment. There was a substantial increase in combined expression of ARGs from day 0 (pre-antibiotic treatment) to day 1 from 74 to 948 TPM, followed by a reduction in expression from day 2 onwards during the course of antibiotic treatment (Figures 1B,C). Expression from day 2 onwards remained lower than at pre-treatment, with the final sample at day 10 having the lowest level of expression at 0.4 TPM. This final level of expression was the lowest observed across the sampled kākāpō population.

Before antibiotic treatment, the kākāpō Joe expressed genes that were attributed to 14 AROs, increasing to 45 AROs on the first day of antibiotic treatment. None of the ARGs expressed by Joe were unique to that bird, with all being detected in other members of the sampled kākāpō population. The AROs observed on the first day of antibiotic treatment were associated with a wider variety of resistance mechanisms and antibiotic drug classes than pre-treatment (Figures 1, 2). Similar to the rest of the population, Joe primarily expressed the *Escherichia coli* EF-Tu mutant conferring resistance to Pulvomycin ARO pre-antibiotic treatment. This pattern continued on the first day of antibiotic treatment, increasing substantially, however no further expression of this ARO was detected after day 1. On days 2 and 8, *Escherichia coli* AcrAB-TolC with MarR mutations was primarily observed, as well as AdeF; expression of the latter was so low across the general kākāpō population it is contained within the “other” category (Figure 1). By day 10, only *E. coli* AcrAB-TolC with MarR mutations remained detectable.

### 3.3 The kākāpō virome

#### 3.3.1 Virome of the kākāpō population

**Figure 5:**
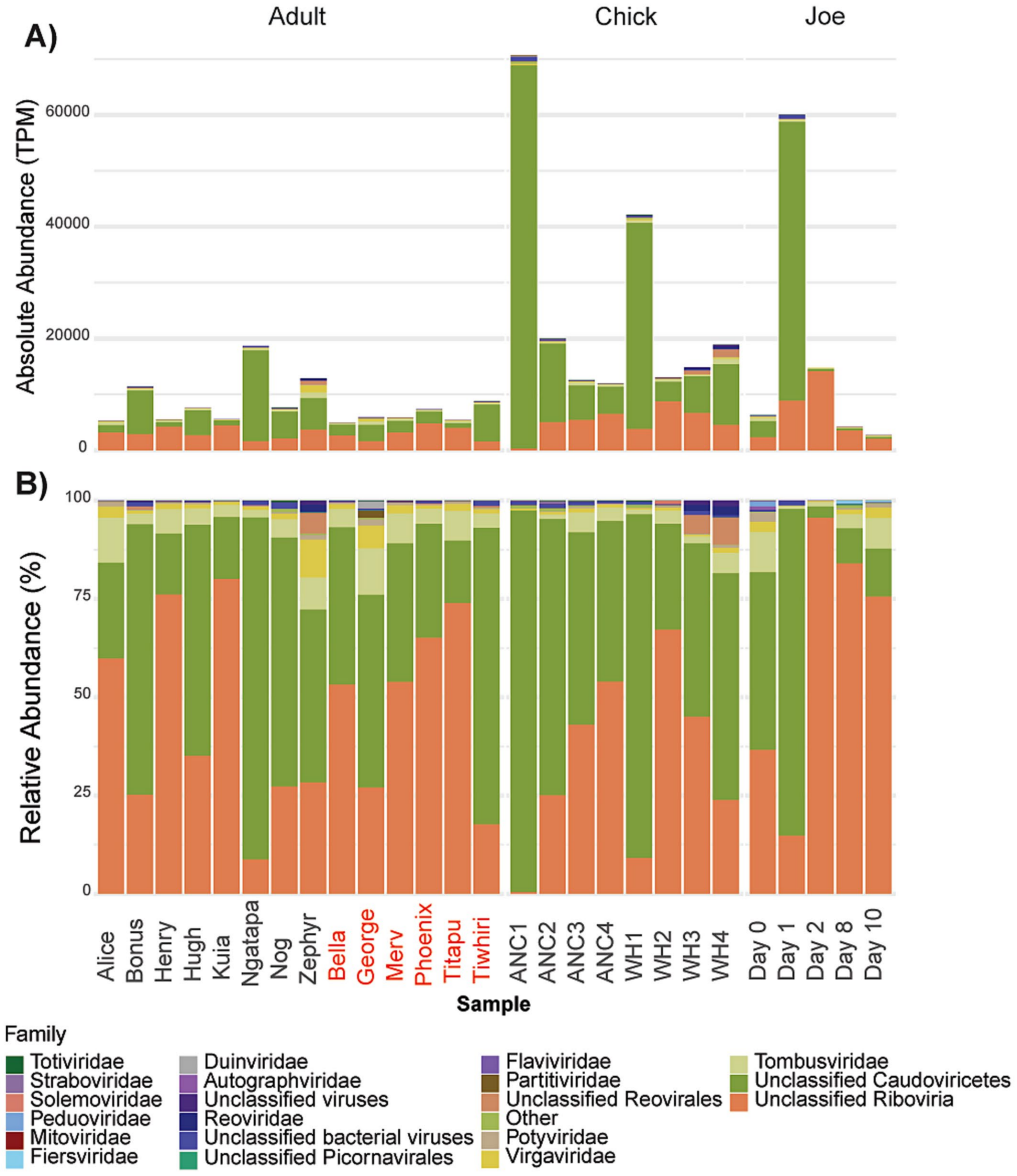
**(A)** Absolute abundance and **(B)** relative abundance of viral transcripts, separated by adults, chicks and antibiotic case-study Joe, where day 0 represents pre-treatment. Adult kākāpō with previous antibiotic exposure indicated by red text. ANC refers to Anchor (Pukenui) Island, WH refers to Whenua Hou (Codfish) Island.

The community of viruses detected was relatively uniform across the sampled adult kākāpō (Figure 5), with Bonus, Ngatapa and Zephyr (kākāpō of various sexes and geographic locations) hosting the highest virus abundances among adults. Chicks appeared to harbour a greater abundance of viruses compared with adults, particularly at the first time point for each island. While there was no significant difference in terms of virus abundance between the two groups of the general adult population, there was a significant difference (Kruskal-Wallis, *p* < 0.05) between chicks and adults of both antibiotic statuses.

Although 13,519 putative RNA vOTUs were initially identified across the entire dataset, after filtering and manual curation of BLAST results to hits matching known viral sequences, only 1828 vOTUs remained. The majority of vOTUs with hits in the nt, nr or RdRp databases that dominated the kākāpō virome could not be classified beyond either the “*Riboviria*” clade of RNA viruses, or the class “*Caudoviricetes*” of bacteriophages (Figure 5). *Caudoviricetes* accounted for the largest proportion of viral sequences in all kākāpō sampled, with 915 vOTUs attributed to this class. Of these, only 139 could be classified to family level, comprising mostly members of the *Autographviridae*, *Duinviridae*, *Fiersviridae*, *Peduoviridae* and *Straboviridae* phage families. Despite a lack of taxonomic information for the 50 vOTUs assigned as unclassified *Riboviria*, the host species from which the matching hits were originally identified appeared to be mainly plants or invertebrates. Among the remaining vOTUs we identified assignments to 74 viral families, of which *Tombusviridae* (single-stranded positive-sense RNA plant viruses) were the most prevalent across the entire sampled kākāpō cohort. *Virgaviridae* and *Potyviridae* (also plant-infecting RNA viruses) were the second and third most common of the *Riboviria* families we could identify. We also found viruses from the *Mitoviridae* and *Totiviridae* families, which are known to infect fungi. No known avian-infecting (or other vertebrate-infecting) viruses were identified in this data set.

There was a strongly significant, positive relationship between expression of ARGs and virus abundance (Spearman’s rank correlation test, *p* < 0.001). This relationship was particularly apparent in the chick samples at the first time point, as well as in the case study samples.

**Figure 6:**
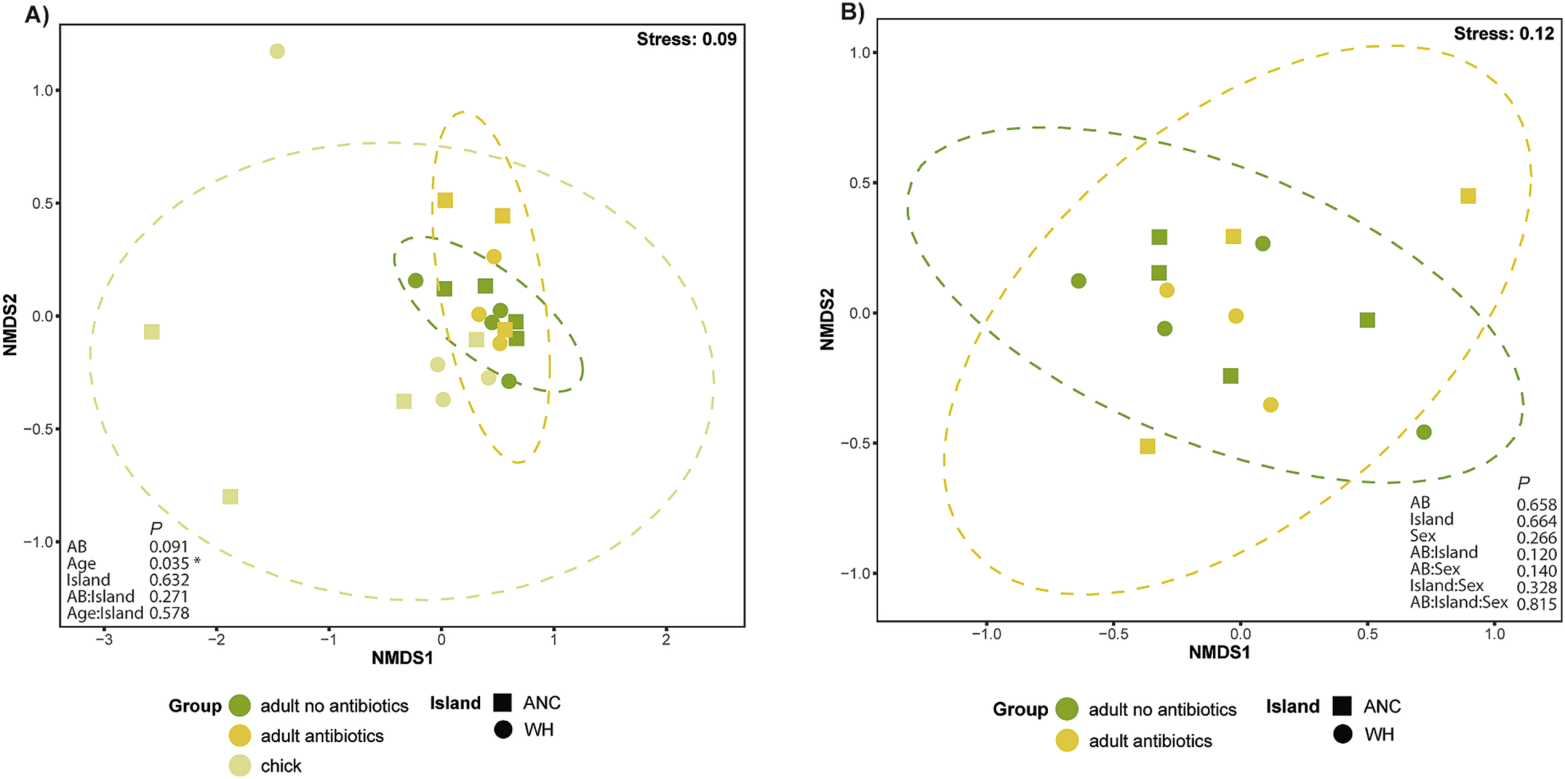
nMDS ordination of the kākāpō virome based on Bray–Curtis dissimilarity. Each point represents a unique RNA library, with adult kākāpō as individuals and chick samples pooled. Ellipses represent variance observed amongst each sampling group with 95% confidence intervals. PERMANOVA results are shown as *p* values **(A)** The adult population by antibiotic exposure, and chick population; **(B)** The adult population by antibiotic exposure.

nMDS ordination conducted on the adult and chick populations showed a tightly clustered group of adults with few outliers. While chicks also generally tended to cluster near the adults, they did have a greater level of dispersion, with three distinct outliers (Figure 6A). PERMANOVA analysis confirmed that age contributed to a significant difference in virome composition (*R*^2^ *=* 0.12, *p* = 0.027), but island and antibiotic status did not, even when considered as interacting factors. Betadisper analysis confirmed that this significant result was not attributable to differences in the dispersion of data. Exclusion of chicks from a further nMDS ordination (Figure 6B) revealed no distinct clusters between the two adult groups, with a subsequent PERMANOVA including age, sex, geographic location and antibiotic status supporting the lack of overall difference in virome composition.

#### 3.3.2 Virome changes with chick developmental stage

Virus abundance was markedly higher at the first time point for chicks at both Anchor and Whenua Hou Islands compared with the final time point (Figure 5). Though the virome of the Anchor chicks appeared to reduce in abundance at each successive time point, this was not the case for the chicks at Whenua Hou which declined between the first and second time point, then had a small increase over time points 3 and 4. While the first time point for Anchor and Whenua Hou was largely dominated by members of *Caudovircetes*, this was even more pronounced in the Anchor chicks. While overall viral reads did decrease, they remained slightly higher for chicks at the fourth time point (15434 TPM) compared to the adults (mean 8048 TPM). Linear mixed modelling was conducted on the chick data to investigate overall temporal trends in total virus abundance. The subsequent ANOVA test showed a significant improvement between the model fit and the null (*X^2^* = 15.078, *p* = 0.001751), indicating that chick age explains a significant amount of the decline in virus abundance.

#### 3.3.3 The virome of an individual kākāpō during antibiotic treatment

Prior to antibiotic treatment, virus abundance in the kākāpō Joe was similar to that of other adult kākāpō, at 6333 TPM. On the first day of antibiotic treatment, measured virus abundance increased to 60064 TPM before decreasing over the remainder of the treatment, with the final sample showing overall viral abundance at 2712 TPM. Virome composition appeared to shift during the treatment period, with *Caudoviricetes* becoming relatively more abundant on the first day of antibiotic treatment but then less so on days 2, 8 and 10 of the treatment (Figure 5B). In terms of relative abundance there was a greater presence of *Peduoviridae* and *Autographviridae* (both *Caudoviricetes*) than seen in the general adult population, however there were still no avian-infecting viruses found in this sample or any viruses that were identified as unique to Joe.

## 4 Discussion

The kākāpō is a critically endangered parrot species with fewer than 250 individuals remaining. They are arguably one of the most intensively managed wild avian species in the world, with antibiotic treatment commonly provided when birds are unwell. Given anecdotal evidence of resistance in cultivated *E. coli* from one kākāpō (not included in this study), there is a need to understand the extent of antibiotic resistance within the current population. We used metatranscriptomics to document the expression of ARGs across kākāpō of varying age, sex, geographic location and, importantly, past antibiotic exposure. Additionally, we were able to further explore the kākāpō virome, reaffirming recent findings for adult kākāpō (West et al., 2025; French et al., 2023, 2025a,b) as well as providing novel insights into the virome of kākāpō chicks.

### 4.1 The kākāpō antibiotic resistome was more diverse and highly expressed in chicks compared with adults

Kākāpō chicks exhibited significantly higher expression of ARGs compared to adults, alongside a greater diversity of ARGs and their associated ARO terms (Figures 1, 3). This complements previous findings from 16S rRNA gene amplicon sequencing which established that the bacterial diversity of the kākāpō chick faecal microbiome significantly decreases with age (West et al., 2022). A more diverse bacterial community in chicks may inherently contribute to the seemingly broader resistome profile, likely due to the wider range of bacterial hosts capable of carrying ARGs. The observed decrease in ARG expression over the first 20 weeks of life may reflect shifts within the bacterial community whereby certain species are initially more transcriptionally active to establish themselves as dominant members of the microbial consortium. This reduction in ARG expression may therefore be associated with stabilisation of the microbiome from a more variable and transient state to the low-diversity adult state, which has been shown to remain robust to changes in anthropogenic diet or geographic location (Perry et al., 2016).

While ARGs are often studied for their clinical relevance in antibiotic drug resistance, and indeed this was an underlying motivation for this study, the expression patterns observed here could also reflect natural ecological processes. Many of the ARGs observed here may serve functions beyond antibiotic drug resistance, such as enhancing bacterial fitness or mediating interactions between microbial community members. This is supported by the widely detected expression of *Escherichia coli* EF-Tu mutants conferring resistance to pulvomycin. Elongation Factor-Thermo Unstable (EF-Tu) is the most abundant protein produced in the bacterial cell, used to translate mRNA transcripts into proteins (Kavaliauskas et al., 2012). Elfamycins are natural products with antibiotic properties produced by *Streptomyces* species that target the process of translation by impairing the function of EF-Tu, by either locking it into the ribosome or covering the binding site for aminoacyl-tRNA. Application of these antibiotics in a clinical setting is limited due to difficulties converting them into therapeutic agents (Prezioso et al., 2017). They have therefore primarily been used in research and laboratory settings in the study of EF-Tu. The presence of elfamycin-resistant EF-Tu mutants within the kākāpō bacterial community is therefore unlikely to have originated from anthropogenic exposure to this antibiotic class, but more likely represents resistance formed by exposure to natural antibiotics.

The implications of H-NS, a global transcriptional repressor protein, being among the most highly expressed ARGs are perhaps of more concern in the context of continued and future antibiotic use in kākāpō. This ARG plays a more indirect role in antibiotic resistance through regulating the expression of other multidrug exporter genes. H-NS is widely distributed within Gram-negative bacteria and is one of the most abundant proteins in the *E. coli* nucleoid. As the microbiomes of many kākāpō are dominated by *E. coli* (Perry et al., 2016; West et al., 2022) the expression of H-NS is perhaps not unexpected.

### 4.2 Absence of avian viruses in the kākāpō virome

For adult kākāpō, our results are similar to those of other recent studies, with the identification of bacteriophages and RNA viruses that seem to infect plants, fungi or invertebrates (West et al., 2025; French et al., 2023, 2025a). While previous studies approached viral discovery from an infectome perspective, we sought here to provide insights into the viromes of chicks in comparison to adults and the development of these viromes over time. Similarly to the expression of ARGs, chicks tended to host a higher viral richness than adults, particularly at the first sampling point where they are between 4 and 8 weeks of age. As the overall abundance of these viruses declined over time, the composition also appeared to shift from being nearly entirely dominated by *Caudovircetes* at the first time point to a relative decrease by the final sampling point, though this effect was more pronounced in chicks from Anchor Island. The change in *Caudoviricetes* abundance was reflective of patterns observed in the ARG profiles. High expression of ARGs, representative of elevated bacterial transcriptional activity, corresponded with a similar pattern of *Caudovircetes* abundance. Thought to be driven by host density (Chevallereau et al., 2022), the lytic and lysogenic life stages of phages may explain some of these observations. As increased bacterial activity may reflect a high population of bacterial hosts in younger chicks, a favourable set of conditions may have enabled *Caudovircetes* phages to enter their lytic cycle and exploit this abundance of hosts. A similar pattern of high *Caudovircetes* abundance was also observed in adult kākāpō that exhibited particularly high levels of ARG expression, such as Ngatapa and Tiwhiri which both had viromes comprising >75% *Caudovircetes.* By comparison, the relative proportion of *Caudoviricetes* to other viruses in Henry, a kākāpō with little ARG expression, was much lower, perhaps indicating lysogenic infection.

Of the named *Riboviria* families in this data set, *Tombusviridae, Virgaviridae* and *Potyviridae* were the most prevalent across the sampled kākāpō. As these are all plant-infecting RNA viruses, the presence of them in any given individual is likely solely related to diet and should be considered as transient.

No known avian-infecting viruses were obtained as part of this data set in either chick or adult kākāpō. This result was not necessarily unexpected as two previous studies (West et al., 2025; French et al., 2025b) were also unable to identify any avian-specific viruses in either healthy or cloacitis-affected adults. These consistent findings across multiple studies are worthy of consideration. As previously discussed (French et al., 2025a), the lack of an apparent virome – in addition to the frequently demonstrated low-diversity microbiome (West et al., 2023)—could be reflective of the genetic bottlenecking events that kākāpō historically underwent. With a loss of ~70–80% of their genetic diversity since the 1800s (Dussex et al., 2021), there is a possibility that this is also reflected in the respective viromes and microbiomes of the kākāpō population. Despite this, however, other species which have undergone similar genetic bottlenecking events, such as the Chatham Island black robin (*Petroica traversi*), do appear to harbour avian viruses (Grimwood et al., 2024). From a disease perspective, it remains possible that a virus responsible for cloacitis (amongst others) exists but is shed from the host organism before the onset of symptoms (and capture of the bird for treatment), thus avoiding detection. Other possibilities include tissue tropism (requiring further investigation into other tissues of the host) or the existence of viruses in kākāpō that are simply too divergent for current sequence similarity searches.

### 4.3 Resistome and virome of a kākāpō undergoing antibiotic treatment: a case study

Our case study of the cloacitis-affected kākāpō under antibiotic treatment suggests that the proliferation of most ARGs does not continue into the latter stages of treatment. Although we detected expression of a protein involved in the multiple antibiotic resistance (Mar) operon (*Escherichia coli* AcrAB-TolC with MarR mutations) on the 10^th^ day of treatment, this was at such low levels that it is not likely to be of concern for this individual. As a repressor protein, MarR is ultimately responsible for regulating the AcrAB efflux pump via the *marRAB* operon, conferring multidrug resistance in enteric bacteria (Sharma et al., 2017). While the current suite of antibiotics used on this kākāpō appears to have remained effective, post-treatment data for this individual are lacking from the current study. We are unable to speculate as to whether the *E. coli* AcrAB-TolC with MarR mutations persisted further or, more generally, whether Joe returned to a similar resistome profile as the other birds and his pre-treatment sample. It should be noted, however, that the antibiotics administered during this study were not Joe’s first exposure and his pre-treatment sample did resemble that of other kākāpō within the population. Further studies into individual kākāpō receiving antibiotic treatment are necessary as this singular case may not be representative of the entire population. The anecdotal evidence of antibiotic resistance in *E. coli* cultivated from a kākāpō not included in this study may, for example, invoke a different set of ARGs that we did not detect in this bird. It is interesting, however, that *E. coli* AcrAB-TolC with MarR mutations was detected as the second most prevalent ARO in two kākāpō previously exposed to antibiotics on Whenua Hou, the island where Joe resides. While this may indicate some level of geographic dependence of expression, it is important to note that kākāpō are regularly moved across the sanctuary islands, and this may not reflect a genuine difference between island populations.

In terms of viruses, a large increase in *Caudoviricetes* was observed on the first day of antibiotic treatment. At the time this sample was taken, it is likely that bacteria had become more transcriptionally active as a stress response invoked by the antibiotics. Prophages integrated into the bacterial host genomes are therefore likely to have been induced to enter the lytic cycle and begin active replication of progeny phage (Howard-Varona et al., 2017). The remaining samples showed a decrease in *Caudoviricetes* relative to other RNA viruses over the course of treatment, which may suggest a lack of bacterial hosts at these time points, thus limiting the ability for *Caudovircetes* to replicate further. This tangentially provides additional evidence for the continued efficacy of antibiotics on this bird. It is also worth noting that viruses themselves can harbour ARGs, as shown in chickens (Wang et al., 2024).

### 4.4 Methodological considerations

While this study may serve as a baseline for “normal” ARG expression in the general kākāpō population, more certainty could be given with an increased number of samples. The low sample size in nearly all microbiological studies of kākāpō (with the notable exception of West et al., 2023) is an inherent challenge of working with such a critically endangered species. While best efforts are made to follow initial experimental design, sampling opportunities are restricted to routine health-checks and monitoring events to minimize bird stress and disturbance, and faecal samples are not necessarily produced on demand. This is further exacerbated by the difficulties of extracting high-quality RNA from the available samples, with failures reducing the number of usable samples and consequently the robustness of the dataset. While more opportunities do arrive for sampling during breeding seasons, where increased human intervention is a necessity, these events take place infrequently (~2–4 years) and conducting sampling should, justifiably, be of lesser priority than activities related directly to successful breeding.

As our study captures the expression, but not overall carriage, of antibiotic resistance genes, our ability to interpret the functional resistome within the context of the broader microbiome remains somewhat limited. In the absence of immediate antibiotic selective pressure, the expression of all ARGs comprising the resistome may not be evident. Future studies incorporating metagenomic sequencing would therefore be beneficial to pair with these findings by identifying both the ARGs actively expressed in kākāpō, as well as those present in the bacterial community that may have the potential to be expressed under clinical antibiotic exposure. Moreover, while it would have been ideal to include parallel analysis of the overall bacterial community (e.g., via 16S rRNA gene sequencing), we were unfortunately unable to obtain DNA for this due to the lack of multiple swabs for every bird in this study.

Finally, choice of reference database for identifying ARGs can potentially influence the conclusions of a study (Papp and Solymosi, 2022). We selected the widely used CARD on the basis that it is not limited to clinical pathogens, includes ARGs from across a broad range of species and environmental contexts, and has detailed functional information including resistance mechanisms (Alcock et al., 2023). Other commonly used databases (e.g., ResFinder) may conceivably lead to different findings. Indeed, ResFinder-based analysis of kākāpō samples in a recent study focused on exudative cloacitis (French et al., 2025b) yielded substantially fewer ARG hits than found in the current study. There is no consensus as to which database is most appropriate (Papp and Solymosi, 2022) and careful consideration needs to be given before embarking on a study.

### 4.5 Implications for kākāpō management

The observed differences between adults of different antibiotic exposure history are interesting. Although differences were not statistically significant (perhaps due in part to weak statistical power from the low sample size), adults on Whenua Hou previously exposed to antibiotics were distinct from kākāpō which had never received antibiotics, regardless of island. The slightly higher number of ARGs and drug resistance classes seen in birds which had previously received antibiotics suggests a potential need for further monitoring and sampling.

Using the individual male kākāpō Joe as a case study indicated that the use of antibiotics appears to remain effective in the kākāpō population. Despite this, consistent monitoring remains important for this critically endangered species. While the use of antibiotics thus far does not appear to have had negative consequences, there nevertheless remains a risk that ARG acquisition may occur via ongoing exposure to veterinary antibiotics or transmission of ARGs from other bird species or even from the humans that care for kākāpō. However, the overall lack of ARG differences with antibiotic history suggests that cautious use of antibiotics can continue.

Ongoing research in our laboratory is focused on cultivation of *Escherichia coli* strains from kākāpō. With the known expression of ARGs identified in this paper, and future whole-genome sequencing of isolates which may identify further ARGs of interest, targeted antibiotic disc-diffusion assays will provide more certainty in the phenotypic expression of ARGs from known constituents of the microbiome. In addition to experimental study on isolates, further work should be undertaken to expand the dataset across a larger proportion of the kākāpō population, particularly those that are receiving antibiotics.

## Data Availability

The data presented in the study are deposited in the NCBI SRA repository, accession number PRJNA1281209.
