# Supplementary material for: Influence of developmental stage on the antibiotic resistome and virome of the critically endangered kākāpō (Strigops habroptilus)
Source: Front Microbiol. 2025 Oct 21;16:1654781. doi: 10.3389/fmicb.2025.1654781 (PMC12582932; doi:10.3389/fmicb.2025.1654781)
Supplement: Supplementary file 1 [file Table_1.docx]

**Table S1:** Adult kākāpō metadata

| **Sample** | **Sample Date** | **Age at sampling (years)** | **Island** | **Sex** | **Antibiotics** |
| --- | --- | --- | --- | --- | --- |
| Alice | 18/10/2022 | unknown (>40) | Codfish/Whenua Hou | F | never |
| Bella | 16/10/2022 | unknown (>40) | Codfish/Whenua Hou | F | previous treatment |
| Bonus | 23/10/2022 | unknown (>40) | Codfish/Whenua Hou | M | never |
| George | 27/11/2022 | 13.7 | Anchor/Pukenui | M | previous treatment |
| Henry | 23/11/2022 | 6.7 | Anchor/Pukenui | M | never |
| Hugh | 11/12/2022 | 6.8 | Anchor/Pukenui | M | never |
| Joe1 | 07/10/2022 | unknown (>40) | Codfish/Whenua Hou | M | pre-treatment (cloacitis) |
| Joe2 | 08/10/2022 | unknown (>40) | Codfish/Whenua Hou | M | during treatment |
| Joe3 | 09/10/2022 | unknown (>40) | Codfish/Whenua Hou | M | during treatment |
| Joe4 | 15/10/2022 | unknown (>40) | Codfish/Whenua Hou | M | during treatment |
| Joe5 | 17/10/2022 | unknown (>40) | Codfish/Whenua Hou | M | during treatment |
| Kuia | 19/11/2022 | 24.7 | Anchor/Pukenui | F | never |
| Merv | 12/10/2022 | unknown (>40) | Codfish/Whenua Hou | M | previous treatment |
| Ngatapa | 22/02/2022 | 0.75 | Anchor/Pukenui | M | never |
| Nog | 23/10/22 | unknown (>40) | Codfish/Whenua Hou | M | never |
| Phoenix | 29/11/2022 | 3.7 | Anchor/Pukenui | F | previous treatment |
| Titapu | 21/10/2022 | 6.7 | Codfish/Whenua Hou | F | previous treatment |
| Tiwhiri | 06/12/2022 | 13.7 | Anchor/Pukenui | F | previous treatment |
| Zephyr | 22/10/2022 | 41.7 | Codfish/Whenua Hou | F | never |
