# Supplementary material for: Influence of developmental stage on the antibiotic resistome and virome of the critically endangered kākāpō (Strigops habroptilus)
Source: Front Microbiol. 2025 Oct 21;16:1654781. doi: 10.3389/fmicb.2025.1654781 (PMC12582932; doi:10.3389/fmicb.2025.1654781)
Supplement: Supplementary file 2 [file Table_2.docx]

**Table S2:** Metadata and pooling information for kākāpō chick samples. The number of individuals contributing to each pool were not the same for every library due to RNA extraction failures and a limited number of cloacal swabs per bird. ANC represents chicks from Anchor/Pukenui Island, WH represents chicks from Whenua Hou/Codfish Island.

| **Sample** | **Kākāpō Name** | **Pool** | **Sample Date** | **Age at sampling (weeks)** | **Island** | **Sex** | **Antibiotics** |
| --- | --- | --- | --- | --- | --- | --- | --- |
| Marian-A2 | Cascade | ANC1 | 16/03/2022 | 5 | Anchor/Pukenui | M | no |
| Marian-A3 | Matilda | ANC1 | 16/03/2022 | 4.8 | Anchor/Pukenui | F | no |
| Waikawa-A1 | Kawa | ANC1 | 18/3/2022 | 6 | Anchor/Pukenui | M | no |
| Waikawa-A2 | Wai | ANC1 | 18/03/2022 | 5.7 | Anchor/Pukenui | M | no |
| Atareta-A2 | Pukaea | ANC2 | 29/04/2022 | 10 | Anchor/Pukenui | M | no |
| Atareta-A3 | Ata Rangi | ANC2 | 29/04/2022 | 9.57 | Anchor/Pukenui | M | no |
| Gertrude-A2 | Paritu | ANC2 | 26/04/2022 | 9.29 | Anchor/Pukenui | F | no |
| Jem-A2 | Awhina | ANC2 | 27/04/2022 | 9 | Anchor/Pukenui | F | no |
| Konini-A2 | Ave | ANC2 | 22/04/2022 | 8.14 | Anchor/Pukenui | F | no |
| Marian-A2 | Cascade | ANC2 | 21/04/2022 | 10.14 | Anchor/Pukenui | M | no |
| Marian-A3 | Matilda | ANC2 | 21/04/2022 | 10 | Anchor/Pukenui | F | no |
| Toitiiti-A1 | Pakiki | ANC2 | 17/04/2022 | 9.29 | Anchor/Pukenui | F | no |
| Atareta-A2 | Pukaea | ANC3 | 27/05/2022 | 14 | Anchor/Pukenui | M | no |
| Atareta-A3 | Ata Rangi | ANC3 | 27/05/2022 | 13.57 | Anchor/Pukenui | M | no |
| Konini-A2 | Ave | ANC3 | 21/05/2022 | 12.2 | Anchor/Pukenui | F | no |
| Marian-A2 | Cascade | ANC3 | 16/05/2022 | 13.7 | Anchor/Pukenui | M | no |
| Marian-A3 | Matilda | ANC3 | 16/05/2022 | 13.57 | Anchor/Pukenui | F | no |
| Toitiiti-A1 | Pakiki | ANC3 | 20/05/2022 | 14 | Anchor/Pukenui | F | no |
| Atareta-A2 | Pukaea | ANC4 | 23/06/2022 | 17.86 | Anchor/Pukenui | M | no |
| Atareta-A3 | Ata Rangi | ANC4 | 23/06/2022 | 17.42 | Anchor/Pukenui | M | no |
| Gertrude-A2 | Paritu | ANC4 | 27/06/2022 | 18 | Anchor/Pukenui | F | no |
| Jem-A2 | Awhina | ANC4 | 02/07/2022 | 18.42 | Anchor/Pukenui | F | no |
| Konini-A2 | Ave | ANC4 | 21/06/2022 | 16.7 | Anchor/Pukenui | F | no |
| Marian-A2 | Cascade | ANC4 | 23/06/2022 | 19.14 | Anchor/Pukenui | M | no |
| Marian-A3 | Matilda | ANC4 | 23/06/2022 | 19 | Anchor/Pukenui | F | no |
| Totiiti-A1 | Pakiki | ANC4 | 20/06/2022 | 18.42 | Anchor/Pukenui | F | no |
| Alice-A2 | Rupi | WH1 | 12/04/2022 | 5.4 | Codfish/Whenua Hou | M | no |
| Solstice-A2 | Valerie | WH1 | 27/03/2022 | 5.4 | Codfish/Whenua Hou | F | no |
| Solstice-A3 | Manakouri | WH1 | 27/03/2022 | 4.9 | Codfish/Whenua Hou | M | no |
| Tohu-A2 | Tangiwai | WH1 | 05/04/2022 | 4.6 | Codfish/Whenua Hou | F | no |
| Margaret-Maree-A1 | Madeline | WH2 | 15/04/2022 | 11.42 | Codfish/Whenua Hou | F | no |
| Pearl-A1 | Matamua | WH2 | 19/04/2022 | 11.14 | Codfish/Whenua Hou | M | no |
| Solstice-A2 | Valerie | WH2 | 24/04/2022 | 9.28 | Codfish/Whenua Hou | F | no |
| Solstice-A3 | Manakouri | WH2 | 24/04/2022 | 8.85 | Codfish/Whenua Hou | M | no |
| Tumeke-A2 | Mason | WH2 | 09/05/2022 | 10.57 | Codfish/Whenua Hou | M | no |
| Zephyr-A2 | Lierz | WH2 | 25/05/2022 | 10.57 | Codfish/Whenua Hou | M | no |
| Alice-A2 | Rupi | WH3 | 09/06/2022 | 13.7 | Codfish/Whenua Hou | M | no |
| Solstice-A2 | Valerie | WH3 | 22/05/2022 | 13.28 | Codfish/Whenua Hou | F | no |
| Solstice-A3 | Manakouri | WH3 | 22/05/2022 | 12.85 | Codfish/Whenua Hou | M | no |
| Titapu-A3 | Bloxham | WH3 | 09/06/2022 | 14 | Codfish/Whenua Hou | M | no |
| Tohu-A2 | Tangiwai | WH3 | 02/06/2022 | 12.8 | Codfish/Whenua Hou | F | no |
| Zephyr-A2 | Lierz | WH3 | 18/06/2022 | 14 | Codfish/Whenua Hou | M | no |
| Pearl-A1 | Matamua | WH4 | 24/05/2022 | 16.1 | Codfish/Whenua Hou | M | no |
| Ra-A3 | Owha | WH4 | 18/06/22 | 16.8 | Codfish/Whenua Hou | F | no |
| Solstice-A2 | Valerie | WH4 | 22/06/2022 | 17.7 | Codfish/Whenua Hou | F | no |
| Solstice-A3 | Manakouri | WH4 | 22/06/22 | 17.28 | Codfish/Whenua Hou | M | no |
| Titapu-A3 | Bloxham | WH4 | 15/07/2022 | 19 | Codfish/Whenua Hou | M | no |
| Tohu-A1 | Kaiako | WH4 | 28/06/2022 | 17 | Codfish/Whenua Hou | M | no |
| Tohu-A2 | Tangiwai | WH4 | 16/07/2022 | 19 | Codfish/Whenua Hou | F | no |
| Zephyr-A2 | Lierz | WH4 | 25/07/2022 | 19.2 | Codfish/Whenua Hou | M | no |
